# Randomized, double‐blind, placebo‐controlled study to assess the efficacy and safety of vortioxetine in Japanese patients with major depressive disorder

**DOI:** 10.1111/pcn.12956

**Published:** 2019-12-18

**Authors:** Takeshi Inoue, Kiyofumi Sasai, Tadayuki Kitagawa, Akira Nishimura, Isao Inada

**Affiliations:** ^1^ Department of Psychiatry Tokyo Medical University Tokyo Japan; ^2^ Takeda Pharmaceutical Company Ltd Osaka Japan

**Keywords:** antidepressive agents, cognition, Japanese, major depressive disorder, vortioxetine

## Abstract

**Aim:**

The burden of major depressive disorder (MDD) in Japan is high. This study aimed to evaluate the efficacy and safety of the multimodal antidepressant vortioxetine in Japanese patients with MDD.

**Methods:**

Japanese patients aged 20–75 years with recurrent MDD and a Montgomery–Åsberg Depression Rating Scale (MADRS) score ≥ 26 were randomized to vortioxetine 10 or 20 mg or placebo in a phase‐3, double‐blind, 8‐week study. The primary end‐point was change in MADRS total score from baseline. Secondary end‐points included MADRS response and remission rates, change in Hamilton Rating Scale for Depression‐17 item (HAM‐D17) score, and other measures of depressive symptoms, including Clinical Global Impression of Severity (CGI‐S), Clinical Global Impression of Improvement (CGI‐I), and Sheehan Disability Scale (SDS). Cognitive function was assessed using Digit Symbol Substitution Test (DSST) score and Perceived Deficits Questionnaire‐5 item (PDQ‐5) score.

**Results:**

Vortioxetine 10 mg (*n* = 165) and 20 mg (*n* = 163) reduced MADRS total score by 2.66 and 3.07 points versus placebo (*n* = 161) after 8 weeks (*P* < 0.01 for each dose), respectively. MADRS response and remission rates were also significantly greater with vortioxetine than with placebo (*P* < 0.05 for both doses). Vortioxetine 10 and 20 mg significantly improved HAM‐D17 score, CGI‐I score, and SDS total score after 8 weeks. PDQ‐5 score was significantly improved in subjects administered vortioxetine, while DSST scores showed no significant difference. Vortioxetine was generally well tolerated.

**Conclusion:**

Vortioxetine at both the 10‐ and 20‐mg/day doses demonstrated robust antidepressant efficacy in Japanese patients with MDD, and was well tolerated over the 8‐week treatment period.

Depressive disorders are the third highest cause of health loss from illness and disability, with major depressive disorder (MDD) affecting approximately 2.2% of individuals worldwide.^1^, ^2^ The prevalence of MDD in Japan is 2.5%, which is similar to the global average, as is the proportion of years lived with disability due to MDD (3.4% vs 3.9% of total years lived with disability).^2^

Japan carries an estimated annual MDD‐related cost burden of US$11 billion (in 2008 US$).^3^ The majority of the burden of disease (US$6.9 billion [63%]) is due to lost workplace productivity, and a further 27% (US$2.5 billion) is due to suicide,^3^ which may be attributed to the symptoms of MDD, including depressive mood, difficulty concentrating/thinking, suicidal ideation, sleep disturbances, and fatigue, as well as impaired mental, social, and cognitive functioning. Of note, both the per‐person and total costs of depression‐related absenteeism in Japan are among the highest in the world; the economic burden also includes the cost of workers presenting for work, but having low productivity because of their condition.^4^ The high rate of suicide is also a major issue in Japan that has been attributed to several sociocultural factors, such as social stress and economic pressure.^4^, ^5^

Japanese guidelines for the treatment of MDD recommend antidepressant therapy, including selective serotonin reuptake inhibitors (SSRI) and serotonin‐norepinephrine reuptake inhibitors, as first‐line treatment options.^6^ However, several unmet needs remain with these therapeutic options for patients with MDD. For example, remission may not be achieved until several interventions have been tried, or recovery may occur slowly because of residual symptoms, such as cognitive symptoms that impair daily functioning.^7^, ^8^, ^9^ In addition, adverse events that may be associated with lower quality of life (QOL) include activation syndrome, sleep disorder, sexual dysfunction, weight gain, and post‐discontinuation symptoms.^10^, ^11^, ^12^, ^13^, ^14^ Japanese psychiatrists are significantly less likely to prescribe antidepressant therapy than their US colleagues, largely because of concerns about tolerability.^15^ Furthermore, data on the efficacy and safety of antidepressant therapies in the Japanese population are limited, so additional studies are required to increase the evidence base in this patient population.^6^

Compared with conventional antidepressant therapies, which selectively inhibit the reuptake of serotonin/norepinephrine with minimal interaction with monoamine receptors, vortioxetine has a differentiated multimodal pharmacological profile, acting as a selective serotonin reuptake inhibitor/serotonin receptor modulator.^16^, ^17^ As a result of vortioxetine's mode of action, several neurotransmitters are modulated beyond serotonin—for example, norepinephrine, dopamine, acetylcholine, glutamate, γ‐aminobutyric acid, and histamine.^18^, ^19^ Vortioxetine was approved by the US Food and Drug Administration for the treatment of adults with MDD in 2013 and has since been approved in more than 80 countries around the world. Indeed, meta‐analyses have found vortioxetine to have one of the most well‐balanced efficacy and safety profiles among antidepressant therapies for patients with MDD.^20^, ^21^ In particular, vortioxetine is associated with a low prevalence of adverse events and improved cognitive function.^22^, ^23^, ^24^, ^25^, ^26^, ^27^

In a phase‐2/3 multinational study including Japanese participants, patients administered vortioxetine had a numerical improvement in Montgomery–Åsberg Depression Rating Scale (MADRS) total score versus placebo in the primary analysis, based on an analysis of covariance (ancova) model using the last observation carried forward (LOCF) method.^28^ This improvement was nominally statistically significant for the 10‐ and 20‐mg doses of vortioxetine when using a mixed‐model repeated measures (MMRM) analysis.^28^ Furthermore, vortioxetine 10 and 20 mg was also associated with a nominally statistically significant increase in response rate and was well tolerated compared with placebo.^28^

Another study of vortioxetine in Japanese patients also reported non‐significant numerical improvements in MADRS total score, although clinically relevant improvements were only achieved in subjects with severe depression (baseline MADRS score ≥ 30) or a recurrent major depressive episode (MDE).^29^ Most treatment‐emergent adverse events (TEAE) were mild to moderate in severity, and comparable with the safety profile of vortioxetine in non‐Japanese patients.^29^

Therefore, this study aimed to further evaluate the efficacy of vortioxetine (10 or 20 mg once daily [QD]) after 8 weeks of treatment in patients with MDD in Japan, as well as the safety and tolerability of this regimen and its effect on cognitive function, using a study protocol that was informed by learnings from these previous studies in Japanese patients.

## Methods

A multicenter, randomized, double‐blind, placebo‐controlled, parallel‐group, phase‐3 study was conducted at 64 sites in Japan from 10 April 2015, until 16 March 2018, investigating the efficacy and safety of vortioxetine in Japanese patients with MDD. This study was conducted in accordance with the principles defined by the Institutional Review Board, Good Clinical Practices and guidelines, and all other applicable regulatory requirements. All patients provided written informed consent prior to enrollment, and investigations were performed in accordance with the ethical principles that have their origin in the Declaration of Helsinki and the International Council on Harmonization tripartite guideline on the ethical principles of Good Clinical Practices. The study was registered on http://ClinicalTrials.gov before enrolling the first patient in the study (http://ClinicalTrials.gov identifier: NCT02389816). The study results are reported here in accordance with the Consolidated Standards of Reporting Trials (CONSORT) guidelines.

Subjects were randomized at a 1:1:1 ratio to vortioxetine 10 mg QD, vortioxetine 20 mg QD, or placebo after completing a 1‐ to 3‐week screening period followed by a single‐blind 1‐week placebo run‐in (Fig. S1). Study drug was administered over an 8‐week double‐blind treatment period, followed by a 4‐week post‐study follow‐up period.

### Inclusion and exclusion criteria

Eligible subjects were aged 20–75 years and had a primary diagnosis of recurrent MDD according to the DSM‐IV‐TR criteria, with the current MDE having lasted 3–12 months (both inclusive). Subjects were required to have an MADRS total score ≥ 26, Hamilton Rating Scale for Depression‐17 item (HAM‐D17) total score ≥ 18, and a Clinical Global Impression of Severity (CGI‐S) score ≥ 4 throughout the screening and placebo run‐in periods, and at the time of entering the double‐blind phase. Female subjects were required to use appropriate contraception from the time of providing informed consent until the end of the safety follow‐up period.

Subjects must not have been diagnosed with any psychiatric disorder, as defined by the DSM‐IV‐TR, including manic, mixed, or hypomanic episode, MDD with psychotic features, schizophrenia or any other psychotic disorder, any substance‐induced mood disorder (except nicotine and caffeine‐related disorders), current or history of clinically significant neurological disorder (including epilepsy), a neurodegenerative disorder (e.g., Alzheimer's disease, Parkinson's disease, multiple sclerosis, or Huntington's disease), or any DSM‐IV‐TR Axis II disorder. However, subjects with symptoms of anxiety remained eligible in the absence of a formal diagnosis of an anxiety disorder. Subjects must not have failed to respond to two or more antidepressants prescribed for ≥ 6 weeks or presented with a positive urine drug‐screening test result.

### End‐points

The primary end‐point was change in MADRS total score after 8 weeks of treatment. Secondary end‐points assessed after 8 weeks of treatment included the proportion of subjects achieving a MADRS response (≥ 50% decrease in the MADRS total score from baseline); proportion of subjects achieving MADRS remission (total score decreased to ≤ 10 from baseline); change from baseline in the HAM‐D17 total score; Clinical Global Impression of Improvement (CGI‐I) score; change from baseline in the CGI‐S score; change from baseline in the Sheehan Disability Scale (SDS) total score; change from baseline in the Digit Symbol Substitution Test (DSST) score; and change from baseline in the Perceived Deficits Questionnaire 5‐item (PDQ‐5) total score. Other end‐points included the change from baseline in the MADRS, HAM‐D, SDS, and PDQ‐5 individual subscale scores.

Safety assessments included adverse events, clinical laboratory tests (serum chemistry, hematology, and urinalysis), vital signs, electrocardiograms, and weight. Suicidal ideation and behavior were also assessed using the Columbia Suicide Severity Rating Scale.

### Statistical analyses

A planned sample size of 480 (160 per group) was determined with the assumption that mean difference of 3.5 and 3.0 for the change from baseline in MADRS total score would provide greater than 80% power to detect the difference by a 2‐sample *t*‐test between each vortioxetine group and the placebo group, and either the vortioxetine 10‐ or 20‐mg group and the placebo group, respectively. The full analysis set (FAS) comprised all subjects who were randomized and received ≥ 1 dose of the study medication in the double‐blind treatment period. The per‐protocol set (PPS) comprised all subjects from the FAS who were evaluable for the primary end‐point and completed the minimum protocol requirements without any major protocol deviations. The safety analysis set comprised all subjects who received ≥ 1 dose of the study medication in the double‐blind treatment period. A list of randomization code was generated and secured by designated personnel, and blinding was maintained by an emergency key management center.

The primary efficacy analysis compared MADRS at Week 8 in the FAS using a MMRM with the change from baseline in the MADRS total score as a dependent variable, and visit, treatment group, visit‐by‐treatment group interaction, and baseline MADRS total score‐by‐visit interaction as fixed effects. Degrees of freedom were adjusted using the Satterthwaite method, and common unstructured covariance matrices were assumed across subjects. Holm adjustment was used to adjust for multiple comparisons. The robustness of the primary efficacy end‐point was assessed by repeating the primary analysis using the PPS.

The change from baseline in the MADRS total score at Week 8 (LOCF) was analyzed using an ancova model with treatment as a fixed effect and the baseline MADRS as a covariate. Odds ratios for MADRS response/remission at Week 8 (LOCF) were compared using a logistic regression model that included MADRS response/remission at Week 8 (LOCF) as a dependent variable, and treatment group and baseline MADRS total score as independent variables. Change from baseline in HAM‐D17, SDS, DSST, and PDQ‐5 total scores at Week 8 (LOCF) was analyzed using an ancova model with treatment as a fixed effect and the baseline score as a covariate. CGI‐I and CGI‐S scores at Week 8 (LOCF) were analyzed using an ancova model with treatment as a fixed effect and the baseline CGI score as a covariate. Where individual subscale scores are analyzed (MADRS, HAM‐D17, SDS, and PDQ‐5), descriptive statistics and two‐sided 95% confidence intervals of means are provided for the observed values and the changes from baseline in the single item at each post‐dose visit by treatment group.

## Results

**Figure 1:**
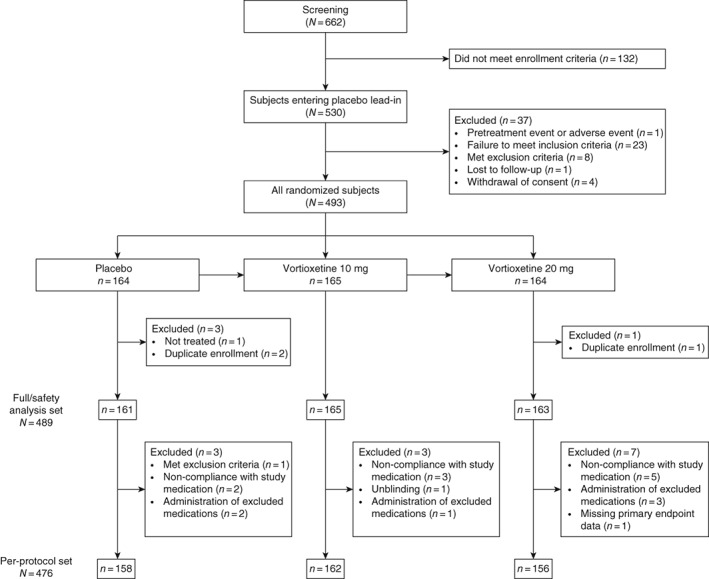
Consolidated Standards of Reporting Trials (CONSORT) diagram.

Of the 662 subjects who provided informed consent, 530 entered the placebo lead‐in period and 493 were randomized (Fig. 1). Of these, 453 subjects completed the double‐blind period; 40 subjects did not complete the double‐blind period, most often because of an adverse event (*n* = 17) or withdrawal of consent (*n* = 14). Study drug was discontinued because of an adverse event in 3.6% and 3.7% of subjects administered vortioxetine 10 and 20 mg, respectively (*n* = 6 each), compared with 2.5% (*n* = 4) administered placebo. One subject randomized to the placebo group did not receive the study treatment because of protocol deviations.

In the total study population, mean age was 40 years, 54.6% of subjects were male, and mean MADRS total score at baseline was 30.6. No appreciable differences in demographic characteristics were observed between treatment groups (Table 1). Mean duration of exposure (range: 53.1–53.6 days) and treatment compliance (> 98%) was comparable between the study arms.

**Table 1:** Patient demographics and baseline characteristics

	Placebo (*n* = 164)	Vortioxetine 10 mg (*n* = 165)	Vortioxetine 20 mg (*n* = 164)	Total (*N* = 493)
Sex, *n* (%)				
Male	92 (56.1)	93 (56.4)	84 (51.2)	269 (54.6)
Female	72 (43.9)	72 (43.6)	80 (48.8)	224 (45.4)
Age, mean years (SD)	39.5 (10.5)	40.0 (10.6)	40.4 (11.3)	40.0 (10.8)
≤ 50, *n* (%)	136 (82.9)	136 (82.4)	126 (76.8)	398 (80.7)
≥ 51, *n* (%)	28 (17.1)	29 (17.6)	38 (23.2)	95 (19.3)
Weight, mean kg (SD)	62.4 (12.2)	62.0 (13.0)	61.5 (12.0)	62.0 (12.4)
BMI, mean kg/m^2^ (SD)	22.4 (3.5)	22.6 (3.6)	22.7 (3.6)	22.6 (3.5)
MADRS total score, mean (SD)	30.5 (3.9)	30.8 (3.7)	30.6 (3.6)	30.6 (3.7)
≤ 30, *n* (%)	94 (57.7)	90 (54.5)	93 (56.7)	277 (56.3)
≥ 31, *n* (%)	69 (42.3)	75 (45.5)	71 (43.3)	215 (43.7)
HAM‐D17 total score, mean (SD)	22.0 (3.2)	22.1 (3.10)	22.2 (3.1)	22.1 (3.1)
CGI‐S score, mean (SD)	4.5 (0.6)	4.5 (0.6)	4.5 (0.6)	4.5 (0.6)
SDS total score, mean (SD)	13.9 (6.2)	14.0 (6.00)	14.8 (5.5)	14.2 (5.9)
DSST score, mean (SD)	60.2 (13.9)	56.8 (15.2)	58.0 (13.7)	58.3 (14.3)
PDQ‐5 score, mean (SD)	9.0 (3.5)	9.5 (3.5)	9.7 (3.5)	9.4 (3.5)

BMI, body mass index; CGI‐S, Clinical Global Impression of Severity; DSST, Digit Symbol Substitution Test; HAM‐D17, Hamilton Depression Rating Scale‐17 items; MADRS, Montgomery–Åsberg Depression Rating Scale; PDQ‐5, Perceived Deficits Questionnaire 5‐item; SDS, Sheehan Disability Scale.

### Efficacy

**Figure 2:**
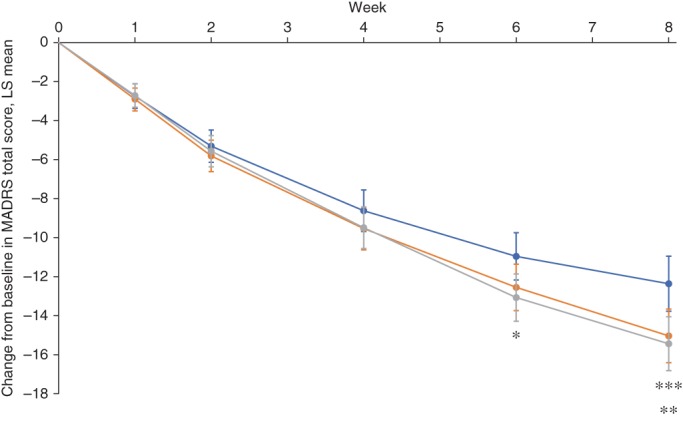
Change from baseline in Montgomery–Åsberg Depression Rating Scale (MADRS) total score over time (full analysis set; mixed‐model repeated measures). Bars represent 95% confidence interval. *P < 0.05 for vortioxetine 20 mg versus placebo. **P < 0.01 for vortioxetine 20 mg versus placebo. ***P < 0.05 for vortioxetine 10 mg versus placebo. (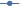) Placebo. (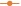) Vortioxetine 10 mg. (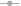) Vortioxetine 20 mg. LS, least squares.

Changes from baseline in MADRS total score at Week 8 were significantly higher in subjects administered vortioxetine 10 and 20 mg than in those administered placebo (Table 2). This difference was consistently observed across the primary analysis and secondary ancova analyses, demonstrating the robustness of the results observed during the primary analysis (Table 2; Fig. 2). Similar results were also observed across subgroup analyses by age, sex, and baseline MADRS total score, except for the vortioxetine 10‐ and 20‐mg arms of the subgroup aged 51 years or older and the vortioxetine arms of the subgroup with MADRS total score at baseline ≥ 31 (see Table S1).

**Table 2:** Change from baseline in MADRS total score after 8 weeks of treatment

	*n*	LS mean (SE)	Difference versus placebo (95% CI)	*P*‐value
FAS (primary analysis; MMRM)				
Placebo	161	−12.37 (0.714)	—	—
Vortioxetine 10 mg	165	−15.03 (0.699)	−2.66 (−4.63, −0.70)	0.0080
Vortioxetine 20 mg	163	−15.45 (0.705)	−3.07 (−5.05, −1.10)	0.0023
PPS (secondary analysis; MMRM)				
Placebo	158	−12.49 (0.712)	—	—
Vortioxetine 10 mg	162	−14.97 (0.698)	−2.48 (−4.44, −0.52)	0.0133
Vortioxetine 20 mg	156	−15.63 (0.708)	−3.14 (−5.12, −1.17)	0.0019
FAS (secondary analysis; ancova using LOCF)				
Placebo	161	−11.83 (0.708)	—	—
Vortioxetine 10 mg	165	−14.52 (0.700)	−2.69 (−5.00, −1.07)	0.0026
Vortioxetine 20 mg	162	−14.86 (0.706)	−3.03 (−4.65, −0.73)	0.0072

ancova, analysis of covariance; CI, confidence interval; FAS, full analysis set; LOCF, last observation carried forward; LS, least squares; MADRS, Montgomery–Åsberg Depression Rating Scale; MMRM, mixed‐model repeated measures; PPS, per‐protocol set; SE, standard error of the mean.

**Figure 3:**
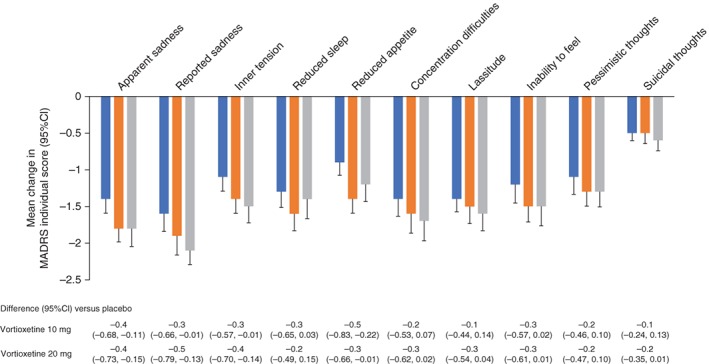
Change from baseline in Montgomery–Åsberg Depression Rating Scale (MADRS) individual scores after 8 weeks (last observation carried forward). (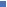) Placebo. (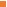) Vortioxetine 10 mg. (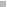) Vortioxetine 20 mg. CI, confidence interval

A significantly higher proportion of subjects administered vortioxetine 10 or 20 mg achieved a MADRS response after 8 weeks compared with patients administered placebo (Table 3). Likewise, MADRS score decreased to ≤ 10 (i.e., remission) in a significantly greater proportion of patients administered either dose of vortioxetine versus placebo (Table 3). Numerically lower MADRS scores versus placebo were also consistently observed across individual scores; no statistical comparisons were conducted for the subscale analyses (Fig. 3).

**Table 3:** MADRS response and remission rates after 8 weeks (FAS; logistic regression analysis using LOCF)

	*n*	Subjects (%)	Odds ratio (95%CI)	*P*‐value
MADRS response				
Placebo	161	59 (36.6)	—	—
Vortioxetine 10 mg	165	79 (47.9)	1.62 (1.04, 2.53)	0.0341
Vortioxetine 20 mg	162	82 (50.6)	1.79 (1.14, 2.80)	0.0110
MADRS remission				
Placebo	161	34 (21.1)	—	—
Vortioxetine 10 mg	165	53 (32.1)	1.84 (1.11, 3.05)	0.0186
Vortioxetine 20 mg	162	50 (30.9)	1.70 (1.02, 2.83)	0.0418

CI, confidence interval; FAS, full analysis set; LOCF, last observation carried forward; MADRS, Montgomery–Åsberg Depression Rating Scale.

**Figure 4:**
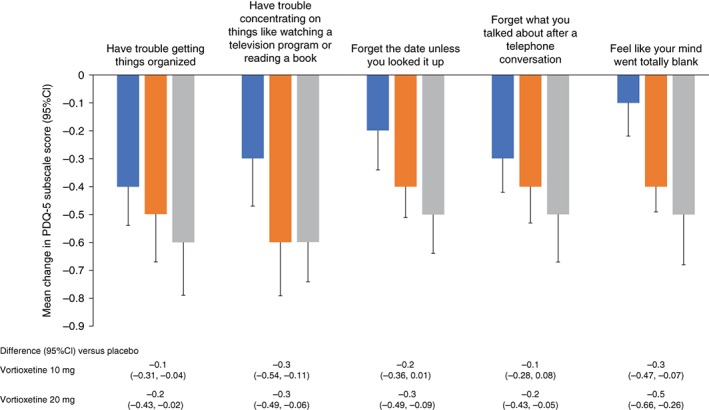
Change from baseline in Perceived Deficits Questionnaire 5‐item (PDQ‐5) subscale scores after 8 weeks (last observation carried forward). (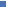) Placebo. (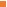) Vortioxetine 10 mg. (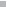) Vortioxetine 20 mg. CI, confidence interval.

Vortioxetine 10 and 20 mg was also consistently associated with significant improvements in the secondary depression‐related end‐points of HAM‐D17 score, CGI‐I score, and SDS total score after 8 weeks (Table 4). Benefits reflected in HAM‐D17 scores were largely related to improvements in depressed mood, work and activities, feelings of guilt, and anxiety (psychic) scores (Fig. S2), while improvements in SDS scores were largely related to reduced symptom‐related disruption of work/school, social life, and family life/home responsibilities (Fig. S3). A significant improvement in CGI‐S score was observed in subjects administered vortioxetine 20 mg, but this did not reach statistical significance for the 10 mg dose (*P* = 0.0609). No significant difference was observed in DSST total score, an objective measure of cognitive function, despite a significant improvement in individual perceptions of cognitive function as assessed by the subjective patient‐reported PDQ‐5 score, with a similar trend observed with the PDQ‐5 subscales (Table 4; Fig. 4).

**Table 4:** Secondary efficacy end‐points after 8 weeks (FAS; ancova using LOCF)

Treatment group	*n*	LS mean (SE)	Difference versus placebo (95%CI)	*P*‐value
HAM‐D17				
Placebo	153	−8.38 (0.53)	—	—
Vortioxetine 10 mg	163	−10.19 (0.52)	−1.81 (−3.29, −0.33)	0.0165
Vortioxetine 20 mg	158	−10.17 (0.53)	−1.79 (−3.28, −0.30)	0.0190
CGI‐I				
Placebo	161	2.77 (0.09)	—	—
Vortioxetine 10 mg	165	2.42 (0.08)	−0.36 (−0.59, −0.12)	0.0031
Vortioxetine 20 mg	162	2.38 (0.09)	−0.39 (−0.63, −0.16)	0.0011
CGI‐S				
Placebo	161	−1.19 (0.09)	—	—
Vortioxetine 10 mg	165	−1.42 (0.09)	−0.23 (−0.47, 0.01)	0.0609
Vortioxetine 20 mg	162	−1.48 (0.09)	−0.29 (−0.54, −0.05)	0.0179
SDS				
Placebo	153	−4.43 (0.44)	—	—
Vortioxetine 10 mg	163	−4.20 (0.43)	−1.34 (−2.56, −0.12)	0.0311
Vortioxetine 20 mg	158	−2.85 (0.45)	−1.57 (−2.81, −0.34)	0.0126
DSST				
Placebo	161	4.92 (0.63)	—	—
Vortioxetine 10 mg	163	4.13 (0.63)	−0.79 (−2.54, 0.97)	0.3793
Vortioxetine 20 mg	162	4.80 (0.63)	−0.11 (−1.86, 1.64)	0.9011
PDQ‐5				
Placebo	161	−1.41 (0.23)	—	—
Vortioxetine 10 mg	165	−2.28 (0.23)	−0.87 (−1.51, −0.22)	0.0089
Vortioxetine 20 mg	162	−2.69 (0.23)	−1.27 (−1.92, −0.62)	0.0001

ancova, analysis of covariance; CGI‐I, Clinical Global Impression of Improvement; CGI‐S, Clinical Global Impression of Severity; CI, confidence interval; DSST, Digit Symbol Substitution Test; FAS, full analysis set; HAM‐D17, Hamilton Depression Rating Scale‐17 items; LOCF, last observation carried forward; LS, least squares; PDQ‐5, Perceived Deficits Questionnaire 5‐item; SDS, Sheehan Disability Scale; SE, standard error of the mean.

### Safety

Vortioxetine was generally well tolerated, with most TEAE being mild or moderate in intensity (Table 5). Nausea, somnolence, and vomiting occurred in ≥ 5% of subjects in the vortioxetine 10‐ and 20‐mg groups. Nasopharyngitis was the only TEAE observed in ≥ 5% of subjects in all groups. All TEAE were mild or moderate in intensity, except for three subjects reporting severe TEAE (altered state of consciousness for one subject administered vortioxetine 10 mg and subarachnoid hemorrhage and cerebral hemorrhage in subjects administered vortioxetine 20 mg [*n* = 1 each]). Approximately 25% of patients reported a TEAE in the first week of study drug administration, in particular, nausea, vomiting, or somnolence, decreasing to approximately 10% of patients during any one of Weeks 2 to 8 (see Fig. S4).

**Table 5:** Overall incidence of adverse events (safety analysis set)

	Placebo (*n* = 161)	Vortioxetine 10 mg (*n* = 165)	Vortioxetine 20 mg (*n* = 163)	Total (*N* = 489)
Subjects with TEAE (%)	75 (46.6)	83 (50.3)	89 (54.6)	247 (50.5)
All TEAE, number of events	104	121	153	378
Relationship to study medication, number of events (%)				
Related	33 (31.7)	76 (62.8)	85 (55.6)	194 (51.3)
Not related	71 (68.3)	45 (37.2)	68 (63.4)	184 (48.7)
Intensity, number of events (%)				
Mild	90 (87.5)	110 (90.9)	140 (91.5)	340 (89.9)
Moderate	14 (12.5)	10 (8.3)	11 (7.2)	35 (9.3)
Severe	0	1 (0.8)	2 (1.3)	3 (0.8)
Death, *n* (%)	0	0	2 (1.2)	2 (0.4)
Serious TEAE, *n* (%)	1 (0.6)	1 (0.6)	3 (1.8)	5 (1.0)
TEAE leading to study medication discontinuation, *n* (%)	4 (2.5)	6 (3.6)	6 (3.7)	16 (3.3)
TEAE in ≥ 5% of subjects, *n* (%)				
Nasopharyngitis	26 (16.1)	23 (13.9)	21 (12.9)	70 (14.3)
Nausea	1 (0.6)	21 (12.7)	25 (15.3)	47 (9.6)
Somnolence	6 (3.7)	7 (4.2)	11 (6.7)	24 (4.9)
Vomiting	0	9 (5.5)	6 (3.7)	15 (3.1)

TEAE, treatment‐emergent adverse event.

Skin and allergic reactions (urticaria, pruritus, dermatitis, rash) were reported by three and six subjects in the vortioxetine 10‐ and 20‐mg groups, respectively, but did not lead to treatment discontinuation. No patients randomized to placebo reported skin or allergic reactions. No subjects experienced a hepatic disorder or overdose.

Vomiting was the TEAE leading to discontinuation in three patients in each of the vortioxetine arms. Insomnia, altered state of consciousness, and headache were also reported as TEAE leading to discontinuation in a single subject each in the vortioxetine 10‐mg arm, and abdominal discomfort, nausea, and subarachnoid hemorrhage in single subjects in the 20‐mg arm. Subjects in the placebo arm discontinued because of nasopharyngitis, headache, akathisia, and sleep attacks (*n* = 1 each).

Serious adverse events occurred in five subjects, with one event being considered by the investigators as likely to be treatment‐related (a fatal cerebral hemorrhage in a 42‐year‐old subject randomized to vortioxetine 20 mg during Week 5 of treatment). One other 65‐year‐old subject administered vortioxetine 20 mg died during the course of the study because of a subarachnoid hemorrhage that was considered to be unrelated to the study drug. Other serious adverse events not attributed to the study drug included altered state of consciousness in a subject administered vortioxetine 10 mg, an anesthetic complication in a subject administered vortioxetine 20 mg, and nephrolithiasis in a subject administered placebo.

No increase in the incidence of suicidal ideation and behavior was observed in the vortioxetine groups compared with the placebo group. No clinically relevant changes in hematology and blood chemistry, vital signs, bodyweight, or electrocardiogram readings were observed in subjects randomized to vortioxetine versus placebo.

## Discussion

Vortioxetine 10 and 20 mg significantly improved MADRS total score after 8 weeks of treatment in Japanese patients with MDD. The improved MADRS total score was paralleled by increased response and remission rates among subjects randomized to either vortioxetine arm compared with placebo and significantly improved outcomes across all secondary measures of depressive symptoms, except for CGI‐S, which only reached statistical significance at the 20‐mg dose. Furthermore, vortioxetine was well tolerated, with adverse events generally being mild or moderate in intensity.

The efficacy of vortioxetine has previously been established in a number of randomized controlled trials in patients with MDD.^30^ In particular, efficacy is largely dose‐dependent and has been consistently observed in studies around the world.^30^ Regardless, vortioxetine treatment significantly increased patients' chances for achieving remission within 8 weeks in this study. Such favorable results are associated with a reduced risk of relapse, psychiatric hospitalization, and suicide, as well as lower health‐care resource utilization.^31^ The 6‐ to 8‐week time to onset of a statistically significant improvement in MADRS score observed in this study is also consistent with the onset of action observed in other studies of vortioxetine.^32^

The safety profile of vortioxetine in this study is consistent with other studies in both Japanese and non‐Japanese patients; the most common adverse events were gastrointestinal in nature with participants reporting an increased prevalence of nausea and vomiting compared with placebo, although the adverse events were generally mild or moderate in intensity.^22^, ^28^, ^29^, ^33^ The prevalence of somnolence was low and also consistent with other studies.^22^, ^29^ Vortioxetine has generally been associated with a lower prevalence of TEAE, including sexual dysfunction and TEAE leading to discontinuation, compared with other antidepressants (duloxetine and extended‐release venlafaxine) in head‐to‐head studies.^22^ In this study, TEAE were mostly reported during the first week of therapy, before dissipating, suggesting that the multimodal activity of vortioxetine may result in a favorable tolerability profile. By comparison, persistent somnolence, nausea, and sexual dysfunction are commonly stated as reasons for late discontinuation of SSRI therapy.^34^ Furthermore, suicidal ideation is rare among patients treated with vortioxetine in clinical trials, and no increased risk has been observed versus placebo, which is consistent with the absence of any trend toward an increased Columbia Suicide Severity Rating Scale score in this study.^22^ In addition, although two deaths due to cerebral and subarachnoid hemorrhage were reported in this study, we conducted an extensive data and literature review and found no evidence from this study or other studies of vortioxetine to suggest that vortioxetine is associated with clinically relevant changes in vital signs or blood pressure that could provoke these incidents.^22^

While vortioxetine has been shown to significantly improve cognitive function as measured by the DSST in randomized, controlled clinical trials (the CONNECT and FOCUS studies) that used the DSST score as their primary end‐points, as well as in two meta‐analyses, no significant difference versus placebo was observed in this study.^23^, ^25^, ^26^, ^27^ However, it must be noted that the CONNECT and FOCUS studies included a cognitive function criterion (excluding baseline DSST scores ≥ 70), whereas this study did not.^23^, ^27^ As a result, baseline DSST scores were higher in this study compared with the CONNECT and FOCUS studies (58.3 vs 41.6–43.5), which may have limited the magnitude of and statistical power to detect any improvement due to a ceiling effect limiting the scope of improvement that can be measured.^23^, ^27^ However, in this study, improvement in cognitive symptoms compared with placebo did reach statistical significance using the PDQ‐5 measures.

The results from this randomized, controlled study are supported by other studies of vortioxetine in Asia. For example, in a real‐world study in patients with moderate‐to‐severe depression in Southeast Asia (Malaysia, Philippines, Singapore, and Thailand), a significant improvement in cognitive function, as measured by Perceived Deficits Questionnaire – Depression scores, along with a large decrease in the depression‐related loss of productivity and impairment at work was observed after 3 months of vortioxetine treatment.^35^ Finally, vortioxetine offered a more cost‐effective option than extended‐release venlafaxine in South Korea in 98% of simulations using a decision tree and Markov model, largely due to a more favorable safety profile increasing relative gains in quality‐adjusted life years with treatment.^36^

The multimodal mechanism of action may be responsible for the differing clinical profile of vortioxetine compared with other antidepressants, given that the pathophysiology of MDD is believed to be underpinned by dysfunction across multiple neurotransmitter pathways.^31^ Ongoing dysfunction in individual pathways may also account for a high proportion of patients with MDD continuing to have residual symptoms after achieving remission with antidepressant therapy, most commonly sleep disturbances, appetite/weight disturbances, cognitive symptoms, and fatigue.^31^ Accordingly, a multimodal mechanism of action across multiple neurotransmitter pathways may account for the cognitive benefits observed in patients treated with vortioxetine.^23^, ^27^ Likewise, the rapid onset of action and favorable tolerability profile of vortioxetine may increase the probability of patients remaining on treatment and achieving remission.^31^, ^37^

Generalization of results to the MDD patient population may be limited by the inclusion criteria of the study (e.g., those with recurrent MDD), and the nature of the baseline characteristics of the study population. The impact of previous antidepressant treatments on the propensity for a patient to respond to the study medication may be confounded by individual patient characteristics, such as disease severity and tolerability issues. More than half of the study population did not have severe MDD; this could have influenced the magnitude of any benefit in the MADRS total score compared with other studies enrolling patients with more severe MDD. The relatively low prevalence of severe MDD may have also contributed to the relatively high baseline DSST scores, increasing the risk of a ceiling effect, as previously noted. Furthermore, the short duration of this study does not provide insight into the long‐term safety and efficacy of vortioxetine in Japanese patients; however, this has been investigated elsewhere in patients with a first or recurrent MDE.^29^ In addition, the age‐related subgroup analysis is limited by the low number of older subjects enrolled in the study, particularly subjects aged 65 years or older; the cut‐off criterion of 51 years was necessary to support a statistical analysis by age subgroup.

The study results confirm the efficacy of the multimodal antidepressant vortioxetine 10 and 20 mg in Japanese subjects with MDD, significantly improving outcomes for the primary end‐point of MADRS total score, and the secondary end‐points of proportion of patients achieving a response and remission, compared with placebo. Vortioxetine also improved self‐reported measures of cognitive function, and was well tolerated, demonstrating a safety profile that was consistent with other studies of vortioxetine in patients with MDD.^22^, ^23^, ^24^, ^25^, ^26^, ^27^

## Disclosure statement

Takeshi Inoue served as medical officer of the study. Kiyofumi Sasai, Tadayuki Kitagawa, Akira Nishimura, and Isao Inada are employees of Takeda Pharmaceutical Company Ltd., Osaka, Japan.

## Author contributions

K.S., T.K., A.N., and I.I. contributed to the conception and design of the study. T.I. contributed to acquisition and analysis of data. All authors contributed to the drafting of this manuscript and approved the final draft prior to submission.

## Supporting information


**Figure S1.** Study design.

**Figure S2.** Change from baseline in Hamilton Rating Scale for Depression (HAM‐D) subscale scores after 8 weeks (last observation carried forward [LOCF]).

**Figure S3.** Change from baseline in Sheehan Disability Scale (SDS) subscale scores after 8 weeks (last observation carried forward [LOCF]). CI, confidence interval.

**Figure S4.** Time course of treatment‐emergent adverse event presentation for (A) nausea, (B) vomiting, and (C) somnolence.

**Table S1.** Change from baseline in Montgomery–Åsberg Depression Rating Scale (MADRS) total score after 8 weeks of treatment by age group, sex, and MADRS total score at baseline.
