# Supplementary figures and images for: Randomized, double‐blind, placebo‐controlled study to assess the efficacy and safety of vortioxetine in Japanese patients with major depressive disorder
Source: Psychiatry Clin Neurosci. 2019 Dec 18;74(2):140–8. doi: 10.1111/pcn.12956 (PMC7027855; doi:10.1111/pcn.12956)

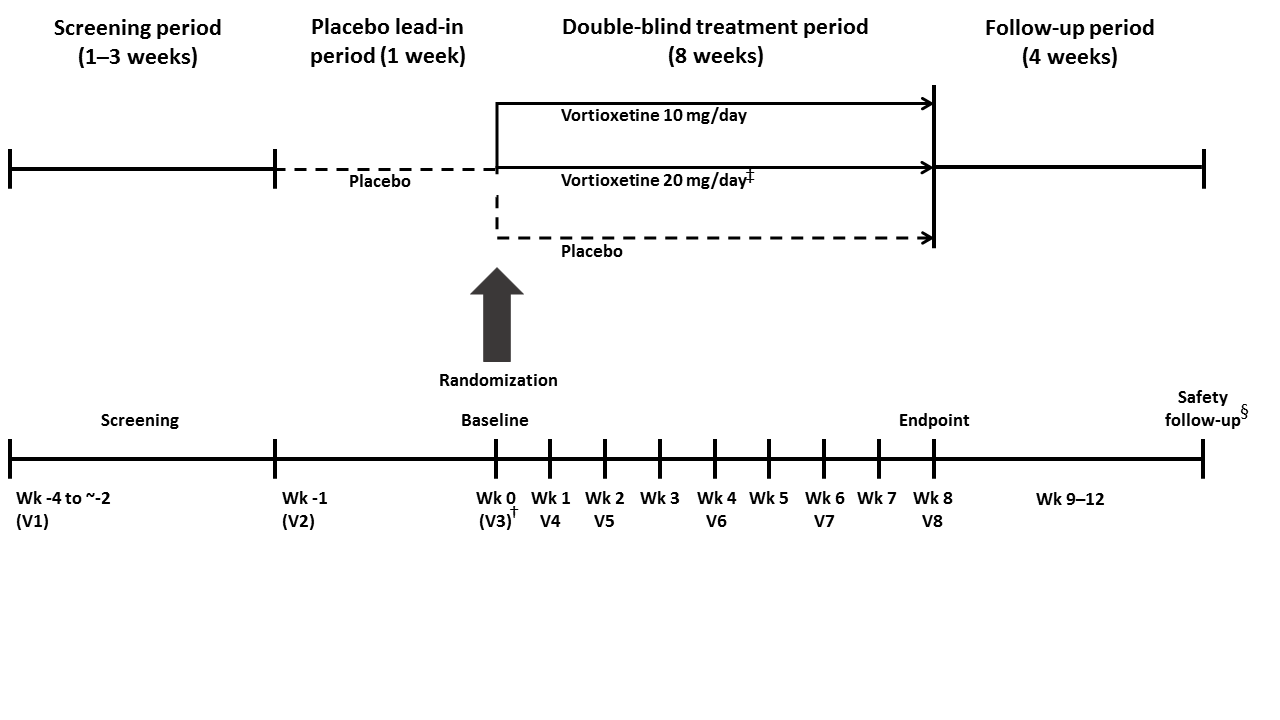

Supplement: Supplementary file 1 — Figure S1. Study design. [file PCN-74-140-s001.tif]

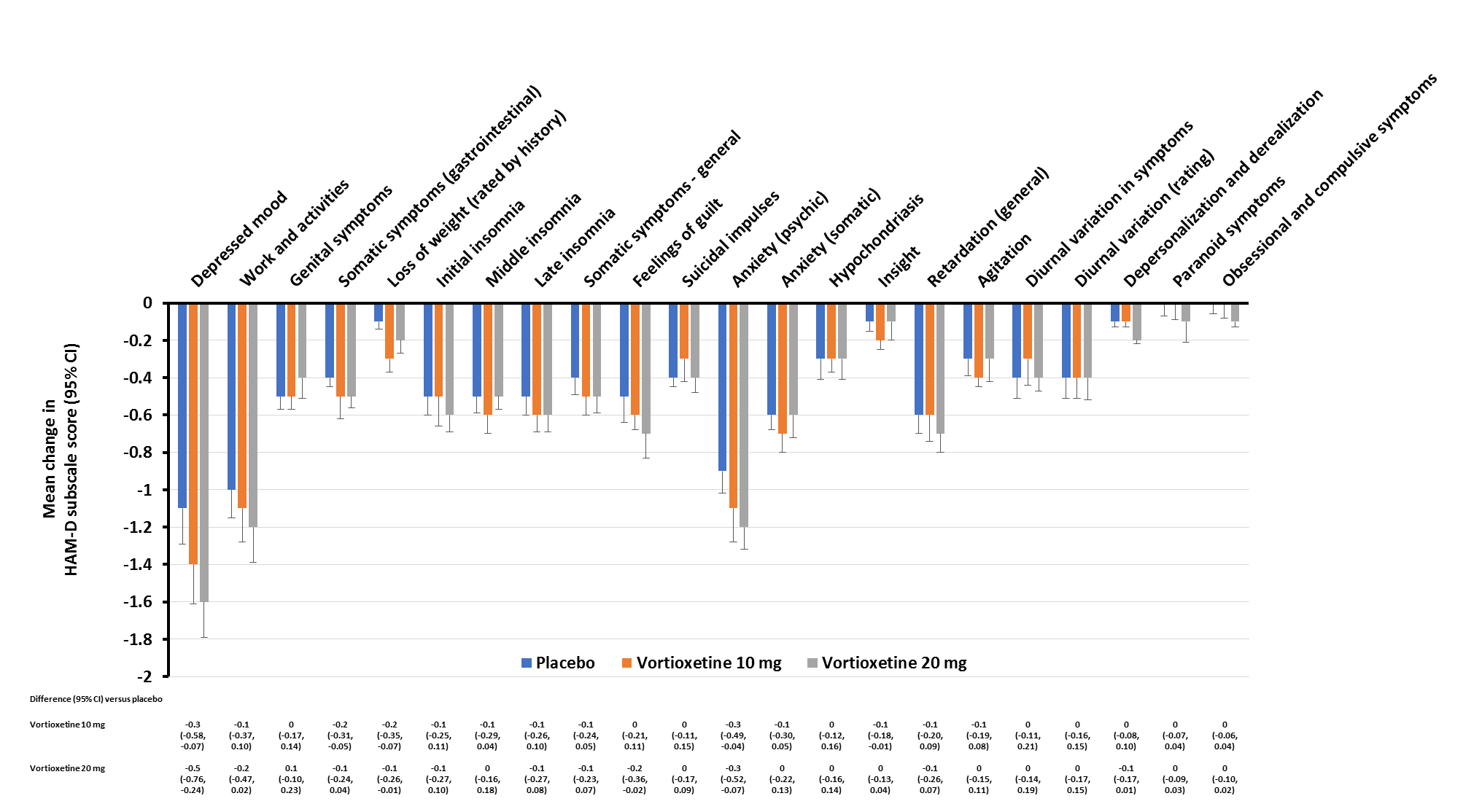

Supplement: Supplementary file 2 — Figure S2. Change from baseline in Hamilton Rating Scale for Depression (HAM‐D) subscale scores after 8 weeks (last observation carried forward [LOCF]). [file PCN-74-140-s002.tif]

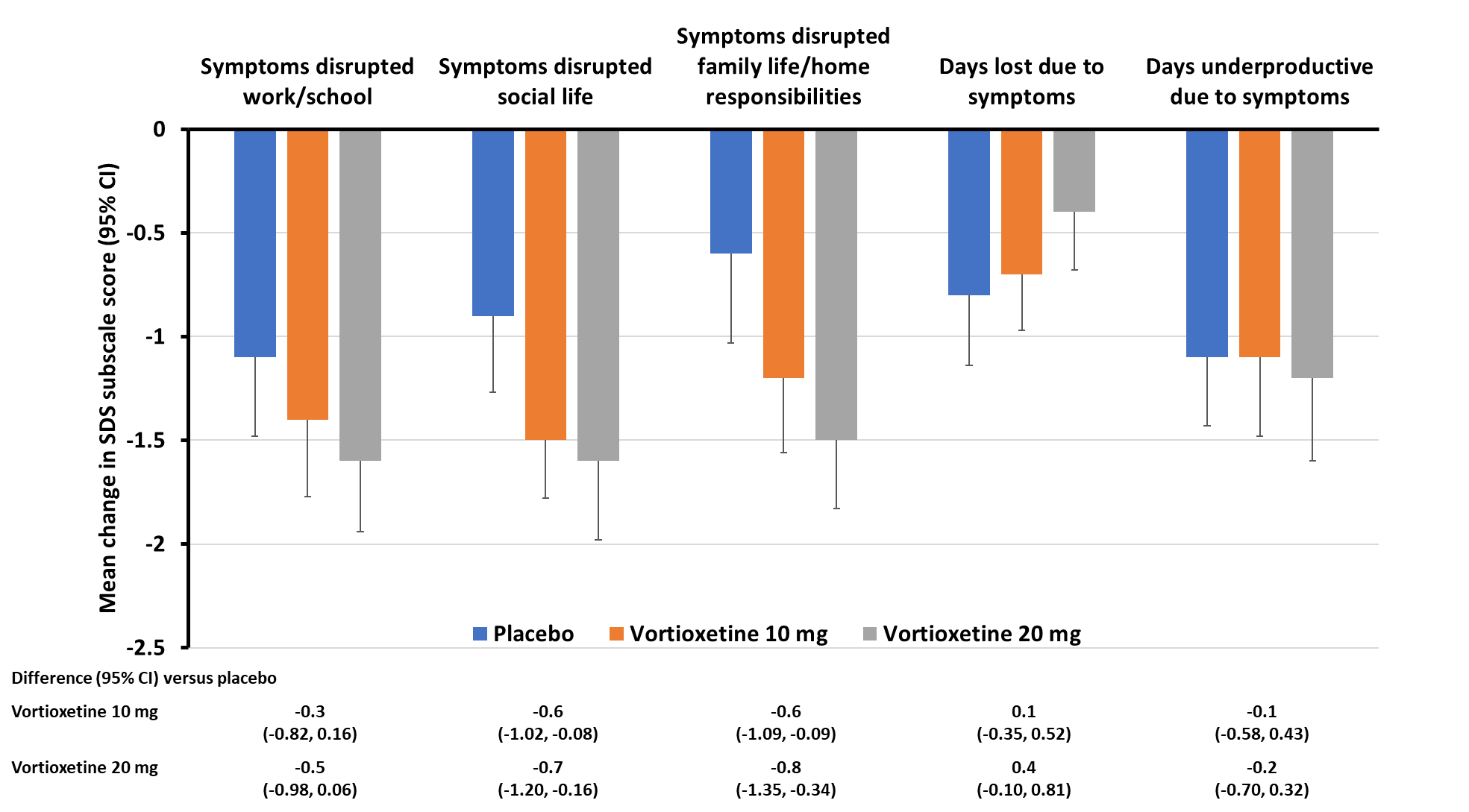

Supplement: Supplementary file 3 — Figure S3. Change from baseline in Sheehan Disability Scale (SDS) subscale scores after 8 weeks (last observation carried forward [LOCF]). CI, confidence interval. [file PCN-74-140-s003.tif]

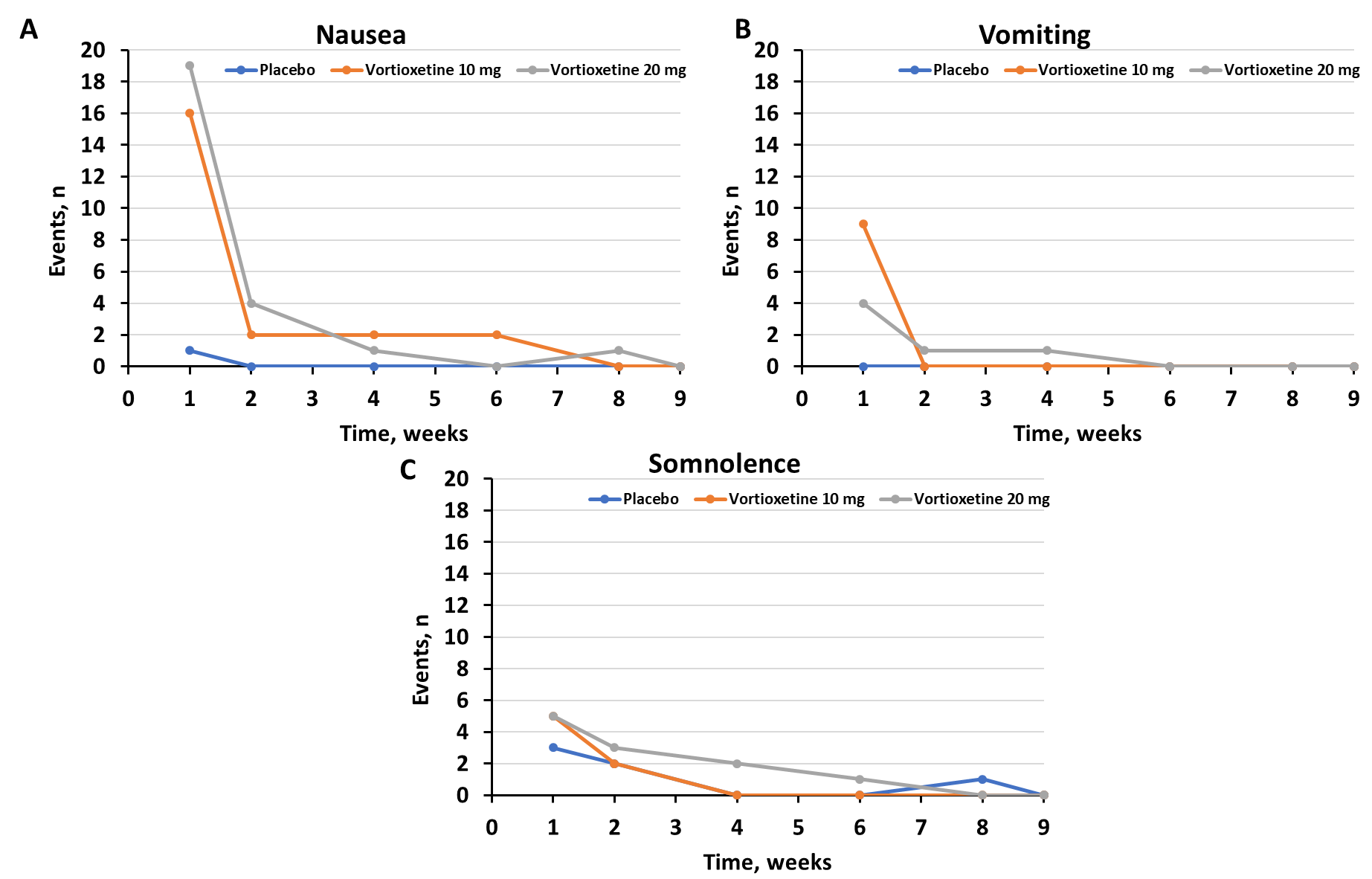

Supplement: Supplementary file 4 — Figure S4. Time course of treatment‐emergent adverse event presentation for (A) nausea, (B) vomiting, and (C) somnolence. [file PCN-74-140-s004.tif]
