# Supplementary material for: Randomized, double‐blind, placebo‐controlled study to assess the efficacy and safety of vortioxetine in Japanese patients with major depressive disorder
Source: Psychiatry Clin Neurosci. 2019 Dec 18;74(2):140–8. doi: 10.1111/pcn.12956 (PMC7027855; doi:10.1111/pcn.12956)
Supplement: Supplementary file 5 — Table S1. Change from baseline in Montgomery–Åsberg Depression Rating Scale (MADRS) total score after 8 weeks of treatment by age group, sex, and MADRS total score at baseline. [file PCN-74-140-s005.docx]

**Supplementary Table 1. Change from baseline in MADRS total score after 8 weeks of treatment by age group, sex, and MADRS total score at baseline**

|  | | | **n** | **LS Mean (SE)** | **Difference of LS mean versus placebo (95% CI)** |
| --- | --- | --- | --- | --- | --- |
| **Age, years** | ≤50 | Placebo  Vortioxetine 10 mg  Vortioxetine 20 mg | 136  136  126 | -12.55 (0.77)  -15.74 (0.75)  -15.96 (0.78) | –  -3.19 (-5.30, -1.08)  -3.41 (-5.56, -1.26) |
|  | ≥51 | Placebo  Vortioxetine 10 mg  Vortioxetine 20 mg | 28  29  38 | -11.58 (1.86)  -11.70 (1.84)  -13.67 (1.61) | –  -0.12 (-5.35, 5.12)  -2.09 (-6.97, 2.79) |
| **Sex** | Male | Placebo  Vortioxetine 10 mg  Vortioxetine 20 mg | 92  93  84 | -12.56 (0.97)  -14.75 (0.95)  -15.51 (1.01) | –  -2.19 (-4.87, 0.48)  -2.95 (-5.71, -0.20) |
|  | Female | Placebo  Vortioxetine 10 mg  Vortioxetine 20 mg | 72  72  80 | -12.15 (1.06)  -15.42 (1.04)  -15.35 (0.99) | –  -3.27 (-6.20, -0.34)  -3.20 (-6.06, -0.33) |
| **MADRS total score at baseline** | ≤30 | Placebo  Vortioxetine 10 mg  Vortioxetine 20 mg | 94  90  93 | -11.39 (0.88)  -14.22 (0.90)  -14.52 (0.87) | –  -2.83 (-5.31, -0.35)  -3.13 (-5.57, -0.68) |
|  | ≥31 | Placebo  Vortioxetine 10 mg  Vortioxetine 20 mg | 69  75  71 | -13.54 (1.20)  -16.13 (1.11)  -16.69 (1.17) | –  -2.60 (-5.82, 0.63)  -3.16 (-6.46, 0.15) |

CI, confidence interval; LS, least squares; MADRS, Montgomery–Åsberg Depression Rating Scale; SE, standard error of the mean.
